# Dynamic circulating tumor DNA indicates pathological benefits of additional neoadjuvant chemoimmunotherapy courses for locally advanced non-small-cell lung cancer patients

**DOI:** 10.3389/fonc.2025.1563315

**Published:** 2025-07-24

**Authors:** Dong Lin, Liang Wu, Pei Wang, Xiaolong Li, Xiaolan Wang, Yiran Cai, Kangli Xiong, Xi Chen, Fu Yang, Wei Huang, Xing Wang, Jiang Fan

**Affiliations:** ^1^ Department of Thoracic Surgery, Shanghai General Hospital, Shanghai Jiao Tong University, School of Medicine, Shanghai, China; ^2^ Department of General Practice, Yangpu Hospital of Tongji University, Shanghai, China; ^3^ Department of Pathology, Shanghai General Hospital, Shanghai Jiao Tong University, School of Medicine, Shanghai, China; ^4^ Geneseeq Research Institute, Nanjing Geneseeq Technology Inc, Nanjing, China; ^5^ Department of Thoracic Surgery, Peking University First Hospital, Beijing, China

**Keywords:** NSCLC, neoadjuvant, immunotherapy, ctDNA-MRD, chemotherapy

## Abstract

**Introduction:**

Few studies have focused on the optimal cycles of neoadjuvant chemoimmunotherapy (NCI). This study introduced minimal residual disease (MRD) based on circulating tumor DNA (ctDNA), and investigated the association between ctDNA-MRD and NCI cycles and pathological response.

**Materials and methods:**

This study was based on a phase III trial (NCT05157776). The patients with IIIA NSCLC without driver genes were given two cycles of NCI (initial two-cycle NCI), after which they were 1:1 randomly assigned to the two-cycle NCI groups (no additional NCI) or four-cycle NCI group (with another two cycles of NCI).

**Results:**

This study involved 13 patients with 28 blood samples. At the start of the study, ctDNA-MRD was detected in 10 out of the 13 patients (77%). The pathologically complete response (pCR) rate was higher in the four-cycle NCI group (3/6, 50%) than in the two-cycle NCI group (1/7, 14.2%). Remarkably, the subgroup that achieved ctDNA-MRD elimination after two cycles and kept elimination after additional two cycles had the highest pCR rate (3/4, 75.0%). Correspondently, the subgroup that did not achieve ctDNA-MRD elimination after two cycles and kept existence after additional two cycles had the lowest pCR rate (0/2, 0%). Generally, in the four-cycle group, a strong correlation between ctDNA-MRD and radiological assessment was observed (5/6, 83.3%).

**Conclusion:**

Patients with locally advanced NSCLC who achieved ctDNA-MRD elimination after two-cycle NCI were more likely to benefit from additional two-cycle NCI, manifesting higher pCR rates. ctDNA-MRD could be a promising tool to determine the optimal cycle of NCI.

**Clinical Trial Registration:**

Clinicaltrial.gov, NCT05157776.

## Introduction

Non-small-cell lung cancer (NSCLC) is one of the most common cancers worldwide (1, 2). Most patients with NSCLC are diagnosed at advanced stages (3). Targeted therapy has proven effective for patients harboring driver gene mutations (4). Immunotherapy has been mainly studied in patients with no driver gene mutations (5). Recently, some studies have demonstrated that neoadjuvant chemoimmunotherapy (NCI) (6, 7) or perioperative chemoimmunotherapy (8) with surgery together provides survival benefits for patients with resectable locally advanced NSCLC.

As to the optimal numbers of neoadjuvant cycles (or preoperative cycles), however, it still needs to be determined (9). In the era of chemotherapy, different cycles of neoadjuvant treatment are applied, such as two, three, and four cycles (10–13). In the era of immunotherapy, the cycles still jump. But a noticeable change is that NCI cycles have become more flexible: for example, the above trials set a maximum number of NCI cycles (6–8). This further calls for the exploration of useful biomarkers of efficacy or toxicity. One recent meta-analysis on esophageal cancer suggested that more than two cycles brought about higher rates of pathological regression together with higher rates of treatment-related adverse events (14). Therefore, there is one question: what is the optimal cycle? And another question: what are the potential biomarkers of the optimal cycles?

In 2021, we initiated a phase III trial (NCT05157776) to investigate the impact of two-cycle NCI versus four-cycle NCI on pathological response in resectable locally advanced NSCLC harboring no driver genes. This trial introduced minimal residual disease (MRD) based on circulating tumor DNA (ctDNA). Previously, ctDNA-MRD has been proven as a valuable biomarker. It was reported to detect tumor recurrence several weeks earlier than radiological methods (15). Recently, some large-scale studies have demonstrated that persistent ctDNA-MRD elimination during chemoradiotherapy could identify clinically cured patients (16, 17). Based on these studies, it may be speculated that ctDNA-MRD is a potentially useful biomarker of the optimal cycles.

Therefore, this study investigated the association between ctDNA-MRD and a.) NCI cycles, and b.) pathological response.

## Materials and methods

### Patient eligibility

**Figure 1:**
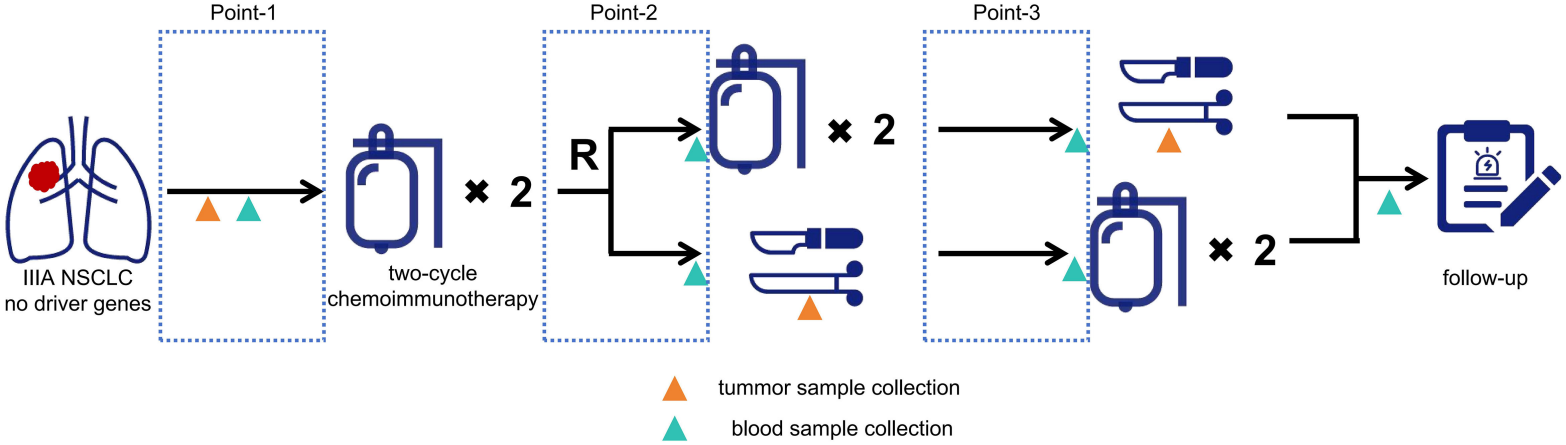
Flowchart of the clinical trial. Point-1, the time interval after disease screening and before the first cycle; Point-2, the time interval after the 2^nd^ cycle and before the 3^rd^ cycle for the 4-cycle group, or the time interval after the 2^nd^ cycle and before surgery for the 2-cycle group; Point-3, the time interval after the 4^th^ cycle and before surgery for the 4-cycle group, or the time interval after surgery and before the postoperative 1 st cycle.

In this ongoing prospective randomized controlled trial (NCT05157776), patients who meet all the following criteria are enrolled: a) diagnosed with resectable IIIA NSCLC, b) without driver genes (such as EGFR, ALK, and ROS-1), and c) not received anti-tumor therapies previously. After obtaining informed consent, the patients were given two cycles of NCI (initial two-cycle NCI), after which they were 1:1 randomly assigned to the two-cycle NCI groups (no additional NCI) or four-cycle NCI group (with another two cycles of NCI) (**Figure 1**). All procedures involving human participants are performed by the 2013 revision of the Declaration of Helsinki (18). This trial was approved by the ethics committee of Shanghai General Hospital (2021-068).

### Clinical and pathological evaluation

**Figure 2:**
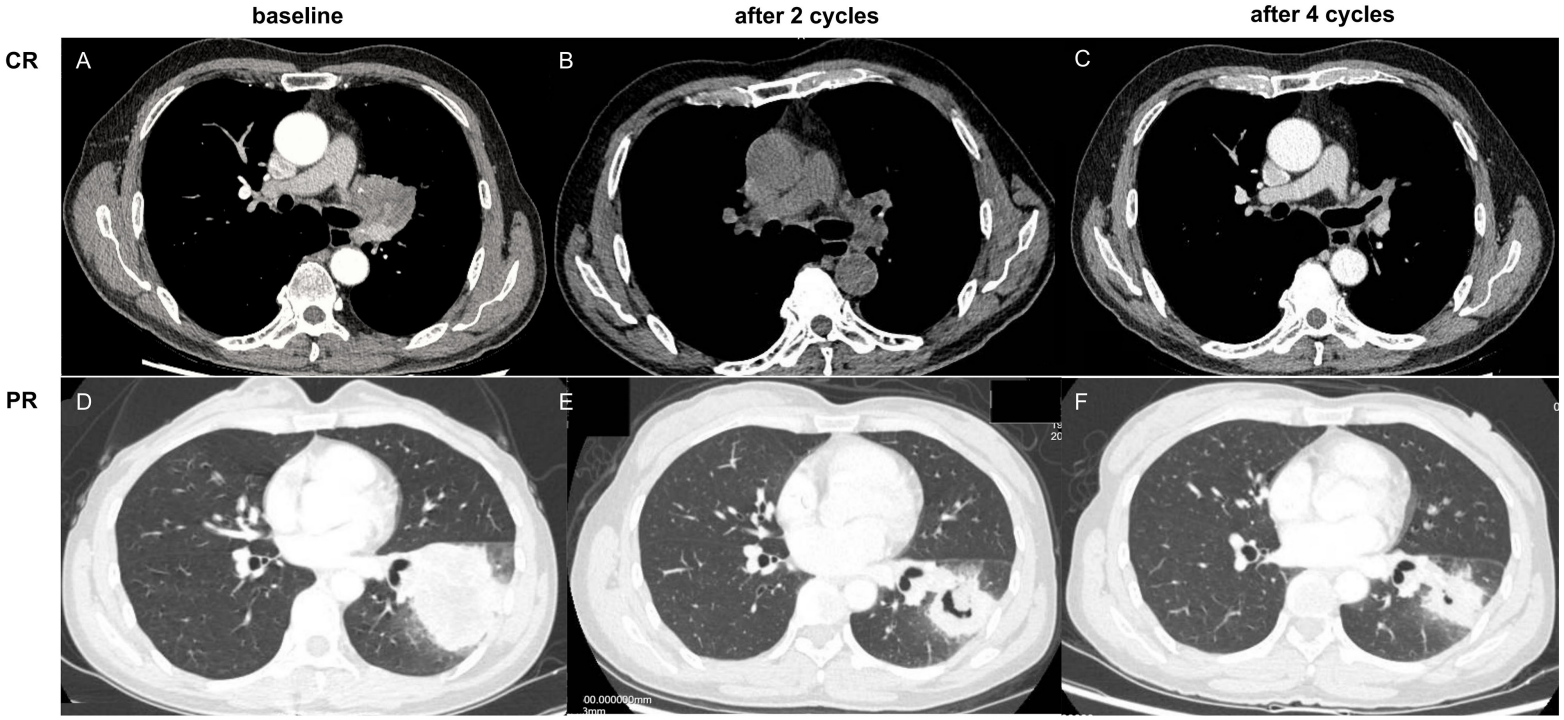
**(A–C)** complete response (CR) based on computed tomography, as notumor issue was found (0.0%); **(D–F)** partial response (PR) based on computed tomography, as tumor regression ≥30%.

**Figure 3:**
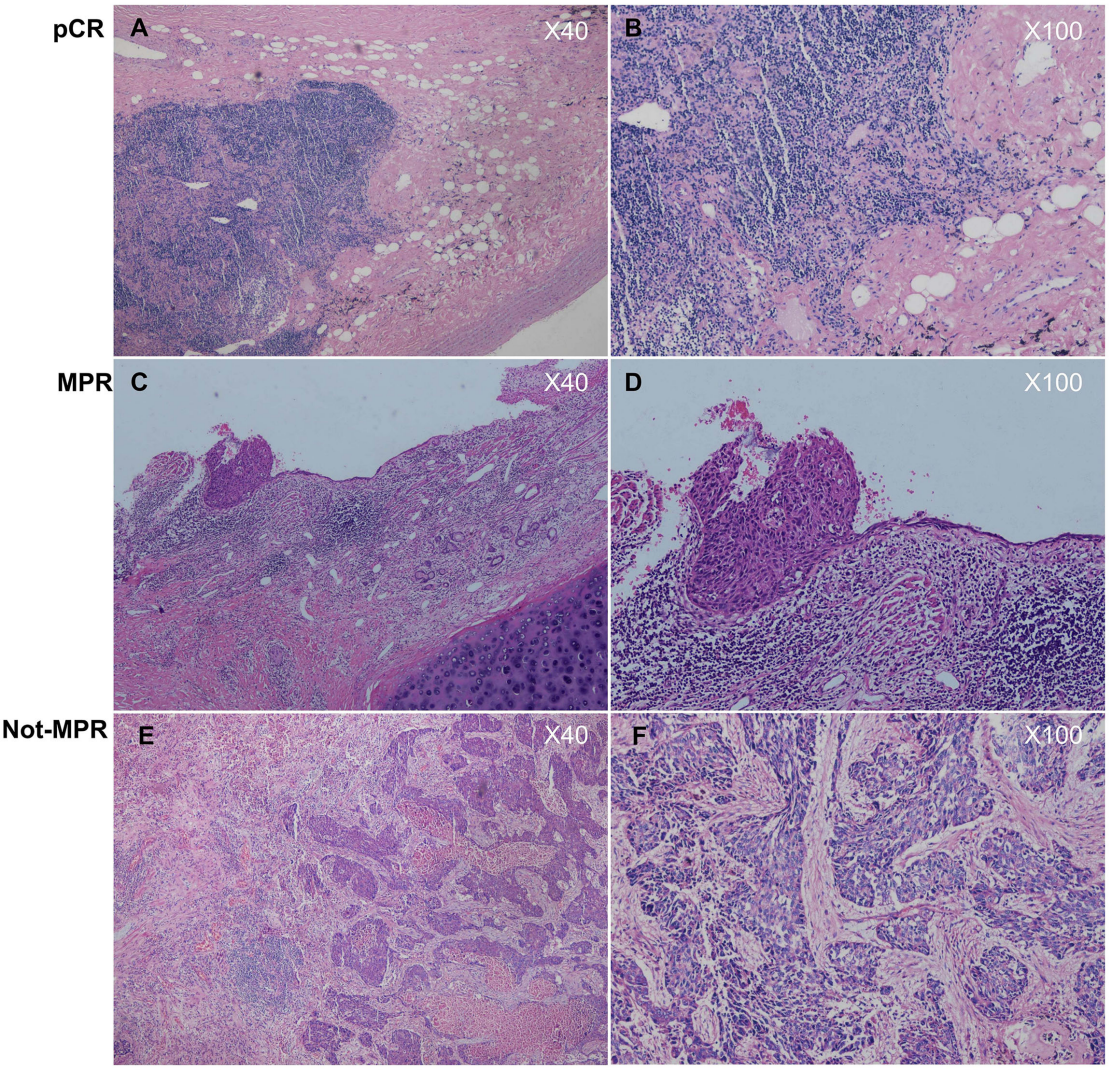
**(A, B)** histologically diagnosed as pathologically complete response (pCR), as no tumor cells were found in both the primary tumor and the lymphnodes; **(C, D)** histologically diagnosed as major pathological regression (MPR), as tumor cells in the primary tumor were ≤10%; **(E, F)** histologically diagnosed as not major pathological regression (Not- MPR), as tumor cells in the primary tumor were >10%.

In this study, the clinical evaluation after NCI was according to iRECIST criteria (19) (**Figure 2**), mainly including complete response (CR), stable disease (SD), and progressive disease (PD). The measurement was based on chest computed tomography. The pathological evaluation was as the following well-recognized criteria (20): major pathological response (MPR) was defined as the presence of no more than 10% of the remaining viable tumor cells in the primary tumor, and pathologically complete response (pCR) was defined as the absence of residual tumor cells in both the primary tumor and the resected lymph nodes (**Figure 3**). The pathological evaluation was independently reviewed by two experienced pathologists (Y.C & X.W). All data were last updated on April 30, 2024.

### ctDNA-MRD detection and validation

#### ctDNA-MRD panel

The MRD of ctDNA in plasma was detected using a targeted next-generation sequencing panel (Vanguard^®^ Nanjing Geneseeq Technology Inc., Nanjing, Jiangsu, China) with a 130-kb coverage on the genome. The ctDNA-MRD panel was developed on the basis of somatic mutational profiles of more than 8000 Chinese NSCLC tumor specimens and nearly 500 lung adenocarcinoma (LUAD) and lung squamous cell carcinoma (LUSC) samples from The Cancer Genome Atlas (http://cancergenome.nih.gov) database. This panel identified somatic single-nucleotide variants (SNVs), insertion deletions (indels), and fusions in 139 crucial cancer-related genes (genelist in **Supplementary File 1**) and identified somatic mutations in over 95% of patients in the total database and over 98% of Chinese patients with lung cancer. A customized library preparation with a bi-barcoding system and ultradeep sequencing approach called Automated Triple Groom Sequencing developed by Nanjing Geneseeq Technology Inc. was applied to the cell-free DNA (cfDNA) samples. To detect low-abundance variation of ctDNA in blood, the following procedures were performed: 1) ultrahigh depth of ~30,000× for plasma samples, 2) a bi-barcode molecular label system used for identifying unique DNA molecules in plasma, 3) a duplex-assisted decoder system used to filter mapping and sequencing errors, and 4) an in-house-developed background database with cfDNA sequencing data from plasma of 100 healthy donors for noise filtering. This panel could detect low-abundance variation of ctDNA in blood. The limit of detection on variant allele frequency (VAF) was 0.01%, as validated with ~30,000× deep sequencing of DNA mixtures of two reference human DNA samples (NA19240 and NA18535).

### Sample collection and processing

Formalin-fixed paraffin-embedded (FFPE) of the tumor samples was collected from patients available at baseline and surgery in this trial. Genomic DNA from tumor tissues and peripheral blood leukocytes was extracted using the QIAamp DNA FFPE Tissue Kit and the DNeasy Blood & Tissue Kit (Qiagen, Hilden, Germany), respectively. At each sampling point (within 24 hours before the planned treatment), 8–10 mL of peripheral blood was collected and the plasma fraction was prepared 2 h after blood collection via centrifugation at 1800×g for 10 min. cfDNA was extracted using the QIAamp Circulating Nucleic Acid Kit (Qiagen, Hilden, Germany). Subsequently, all the extracted DNA was quantified using the Qubit 2.0 Fluorometer and the dsDNA High-Sensitivity Assay Kit (Life Technologies, Eugene, OR, USA) to determine the cfDNA yield. Sequencing libraries were prepared using the KAPA HyperPrep Kit (KAPA Biosystems, Wilmington, MA) according to the manufacturer’s protocol.

### Library preparation and sequencing

Libraries were prepared using the KAPA HyperPrep Kit (KAPA Biosystems, Wilmington, MA) according to the manufacturer’s protocol. For tumor tissue or peripheral blood leukocytes, 1–2 μg of genomic DNA sheared into ~350-bp fragments using a Covaris M220 instrument (Covaris) was subjected to end-repairing, A-tailing, and indexed adapter ligation, followed by size selection using Agencourt AMPure XP beads (Beckman Coulter). Hybridization-based target enrichment was performed using customized xGen Lockdown Probes (Integrated DNA Technologies) targeting 425 cancer-relevant genes (GeneseeqPrime^®^, Nanjing Geneseeq Technology Inc., Nanjing, Jiangsu, China) (genelist in **Supplementary Materials**). For plasma samples, more than 10 ng and up to 50 ng of cfDNA underwent end-repairing, A-tailing, ligation with a customized adapter containing a unique molecular index (UMI), and PCR amplification with primers containing demultiplexing indices. Customized xGen lockdown probes (Integrated DNA Technologies) targeting 139 lung cancer-relevant genes (Vanguard^®^, Nanjing Geneseeq Technology Inc., Nanjing, Jiangsu, China) were used for the hybridization enrichment of plasma libraries. All captured libraries were amplified with Illumina p5 (50 AAT GAT ACG GCG ACC GA 30) and p7 primers (50 CAA GCA GAA GAC GGC ATA CGA GAT 30) in KAPA HiFi HotStart ReadyMix (KAPA Biosystems), followed by qPCR quantification using the KAPA Library Quantification Kit (KAPA Biosystems) and purification using Agencourt AMPure XP beads. The library fragment size was assessed using a 2100 Bioanalyzer (Agilent Technologies). The target-enriched library was then sequenced on the Illumina HiSeq4000 platform according to the manufacturer’s protocol. The average coverage depth was 1142× and 260× for tissue and leukocyte samples, respectively.

### Mutation calling

All sequencing data were analyzed using Trimmomatic (21) for FASTQ file quality control, with low-quality reads (quality reading below 30) or N bases removed. Qualified reads were then mapped to the reference human genome (hg19) using the Burrows–Wheeler Aligner (https://github.com/lh3/bwa/tree/master/bwakit). PCR duplicates were removed using Picard (Broad Institute, MA, USA) following local realignment around known indels and base quality recalibration using the Genome Analysis Toolkit (GATK 3.4.0).

For tissue samples, SNVs and indels were detected using VarScan with default parameters. Genomic fusions were identified by FACTERA (22) with default parameters. Mutations that were observed in ≥20 cancer cases reported in the COSMIC database were defined as hotspots. For hotspot mutations or other mutations, the minimum VAF was set to 1% or 2% and the minimum variant supporting reads were 5 or 6, respectively.

For cfDNA samples, single-strand consensus sequences were aggregated by the UMI system and generated to remove errors from sequencing and PCR. The mean coverage for the single-strand consensus read without PCR duplicates of plasma was ~6146×.The mutation cutoff was 0.2% for cfDNA samples with a minimum of three unique mutant reads. The background noise was further polished by the aforementioned background database. Germline and clonal hematopoiesis-derived mutations were filtered out based on data from normal control samples. Mutations identified as somatic in the matched tumor with a minimum of one unique consensus mutant allele read in plasma were also detected. ctDNA positivity was defined as the presence of one or more mutations identified in the matched tumor sample in ctDNA.

## Results

### Clinical and pathological characteristics of the whole patients

This study included thirteen patients with twenty-eight blood samples, with a median age of 65. Eleven patients were diagnosed with lung squamous cell carcinoma and two with lung adenocarcinoma. Six patients were in the four-cycle NCI group, while seven were in the two-cycle NCI group.

After the completion of NCI and before surgery, 3 (3/13, 23.1%), 1 (1/13,7.7%), and 9 (9/13, 69.2%) patients were radiologically evaluated for CR, SD, and PR, respectively. As to surgical procedures, 6 (6/13, 46.2%), 4 (4/13, 30.8%), and 3 (3/13, 23.1%) patients underwent regular lobectomy, regular sleeve lobectomy, and double sleeve lobectomy, respectively. All patients underwent standard mediastinal lymph node dissection.

After surgery, the pCR rate was higher in the four-cycle NCI group (3/6, 50.0%) than in the two-cycle NCI group (1/7, 14.2%). The MPR rate was also higher in the four-cycle NCI group (5/6, 83.3%) than in the two-cycle NCI group (5/7, 71.4%). According to these outcomes, it seemed that the four-cycle NCI brought about better pathological response than the two-cycle group. These perioperative characteristics of the patients were shown in **Table 1**.

**Table 1:** General characteristics of the enrolled patients.

Case no.	Age	Gender	Smoking	Histology	cStage	Neoadjuvant cycles	Clinical evaluation	ypTNM0	MPR
No.1	66	Male	Ever	Squa	IIIA	4	CR	T0N0 (pCR)	Yes
No.2	40	Female	Never	Squa	IIIA	4	PR	T1cN1	Yes
No.3	70	Male	Ever	Squa	IIIA	2	PR	T0N0 (pCR)	Yes
No.4	57	Male	Ever	Squa	IIIA	4	PR	T1cN1	No
No.5	58	Male	Ever	Squa	IIIA	2	PR	T1aN0	Yes
No.6	55	Male	Ever	Squa	IIIA	2	PR	T1bN0	No
No.7	65	Male	Ever	Adeno	IIIA	2	PR	T1cN1	Yes
No.8	71	Male	Ever	Squa	IIIA	2	PR	T1N0	Yes
No.9	70	Male	Ever	Squa	IIIA	2	PR	T1aN0	Yes
No.10	47	Male	Ever	Adeno	IIIA	2	SD	T1bN1	No
No.11	68	Male	Ever	Squa	IIIA	4	PR	T0N1	Yes
No.12	57	Male	Ever	Squa	IIIA	4	CR	T0N0 (pCR)	Yes
No.13	67	Male	Ever	Squa	IIIA	4	CR	T0N0 (pCR)	Yes

Squa, lung squamous cell carcinoma; Adeno, lung adenocarcinoma; CR, complete regression; PR, partial regression; ypTNM, pathological TNM after neoadjuvant treatment; ypT0N0, namely pathologically complete regression (pCR); MPR, major pathological response.

### ctDNA-MRD detection in two-and four-cycle groups

**Figure 4:**
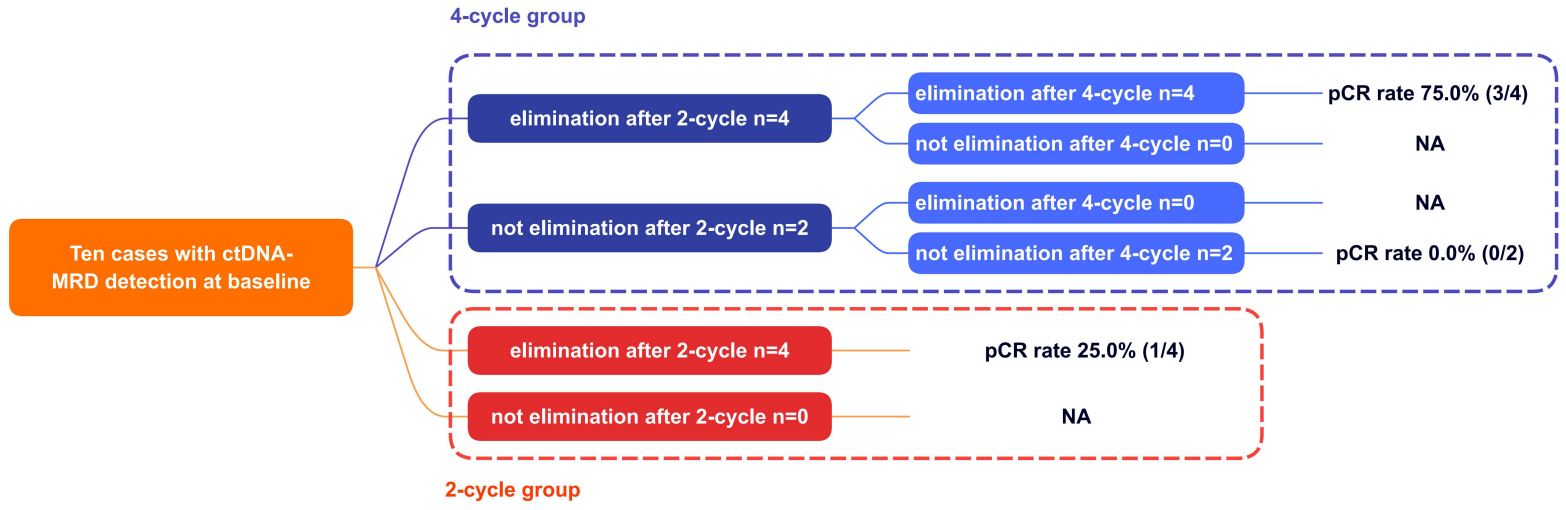
The association between ctDNA changes and pCR. pCR, pathologically complete response; NA, not applicable.

At baseline, ctDNA was detectable in 10 (10/13, 77.0%) patients (except Case No.6, 9, 10). As shown in **Figure 4**, in the two-cycle group, 4 (4/4, 100.0%) patients achieved ctDNA-MRD elimination after two-cycle NCI. This implied that two-cycle NCI was able to make ctDNA-MRD elimination. In the four-cycle NCI group, the outcomes were interesting. Four (4/6, 66.7%) patients achieved ctDNA-MRD elimination after two-cycle NCI; furthermore, all these patients remained ctDNA-MRD elimination after another two-cycle NCI. Finally, this subgroup had the highest pCR rate of 75.0% (3/4). Correspondingly, two (2/6, 33.3%) patients did not achieve ctDNA-MRD elimination after two-cycle NCI; furthermore, both these patients remained ctDNA-MRD existence after another two-cycle NCI. Finally, this subgroup had the lowest pCR rate of 0.0% (0/2). This implied that if pCR were used as a surrogate for survival benefit, the former subgroup might benefit much more from additional NCI than the latter.

### Association between ctDNA-MRD and NCI cycles and pathological response

The above analysis implied a specific association between ctDNA-MRD elimination and NCI cycles and pCR. In this part, **Table 2** showed the ctDNA-MRD dynamics of the ten cases during NCI treatment for further analysis.

**Table 2:** The dynamic ctDNA-MRD and clinical and pathological responses of the ten cases.

Groups	Case no.	Point-1 ctDNA, MaxVAF, % (tumor diameter, mm)	Point-2 ctDNA, MaxVAF, % (tumor diameter, mm)	Point-3 ctDNA, MaxVAF, % (tumor diameter, mm)	Clinical evaluation	ypTNM0	Pathological evaluation
4-Cycle		baseline	before cycle 3	before surgery			
	No.1	7.03 (41)	0 (19)	0 (0)	CR	T0N0	pCR
	No.2	9.23 (67)	0.42 (38)	0.1 (33)	PR	T+N+	MPR
	No.4	1.60 (45)	0.05 (35)	0.32 (16)	PR	T+N+	Not-MPR
	No.11	13.42 (67)	0 (51)	0 (45)	PR	T0N+	MPR
	No.12	0.28 (20)	0 (10)	0 (0)	CR	T0N0	pCR
	No.13	1.8 (35)	0 (0)	0 (0)	CR	T0N0	pCR
2-Cycle		baseline	before surgery	NA			
	No.3	0.27 (45)	0 (20)		PR	T0N0	pCR
	No.5	0.85 (57)	0 (26)		PR	T+N0	MPR
	No.7	0.12 (47)	0 (21)		PR	T+N+	Not-MPR
	No.8	0.58 (48)	0 (13)		PR	T1N0	MPR

4-Cycle, the 4-Cycle group; 2-Cycle, the 2-Cycle group; ctDNA, circulating tumor DNA; MaxVAF, maximal tumor somatic variant allelic frequency; mm, millimeters; NA, not applicable; CR, complete regression; PR, partial regression; ypTNM, pathological TNM after neoadjuvant treatment; T+, with tumor cell residual in the primary tumor; N+, with tumor cell residual in the dissected nodes; ypT0N0, equal to pCR, namely pathologically complete regression; MPR, major pathological regression, namely ≤10% tumor cell residual in the primary tumor.

As two-cycle NCI was enough to make ctDNA-MRD elimination (like Cases No.1, 11, 12, 13; Cases No.3, 5, 7, 8), what another two-cycle NCI would make for those not achieved ctDNA-MRD elimination after the initial two-cycle NCI? As indicated by Cases No.2 and 4, another two-cycle NCI still did not make ctDNA-MRD elimination. Thus, it could be presumed that additional NCI would possibly not bring about ctDNA-MRD elimination when ctDNA-MRD elimination did not happen after the initial NCI.

It was observed that ctDNA-MRD elimination would not always be followed by pCR (like Case No.11 and Case No.5, 7, and 8). However, it was remarkable that all the pCR patients had achieved ctDNA-MRD elimination (like Case No.1, 12, and 13 and Case No.3). These seemed to indicate that ctDNA elimination was a necessary but not sufficient condition for pCR.

When all these findings were taken into consideration, it could be suspected: when the patients who did not achieve ctDNA-MRD elimination after initial NCI received additional NCI, would they subsequently achieve ctDNA-MRD elimination, and finally achieve pCR?

### Association between ctDNA-MRD and tumor diameters during NCI cycles

Clinical evaluation based on tumor diameters was still the mainstream. As shown in **Table 2**, it was observed in the two-cycle NCI group that ctDNA-MRD dynamics were consistent with tumor diameter changes before and after NCI. The accordance rate of the trends was recorded as 100.0% (4/4).

**Figure 5:**
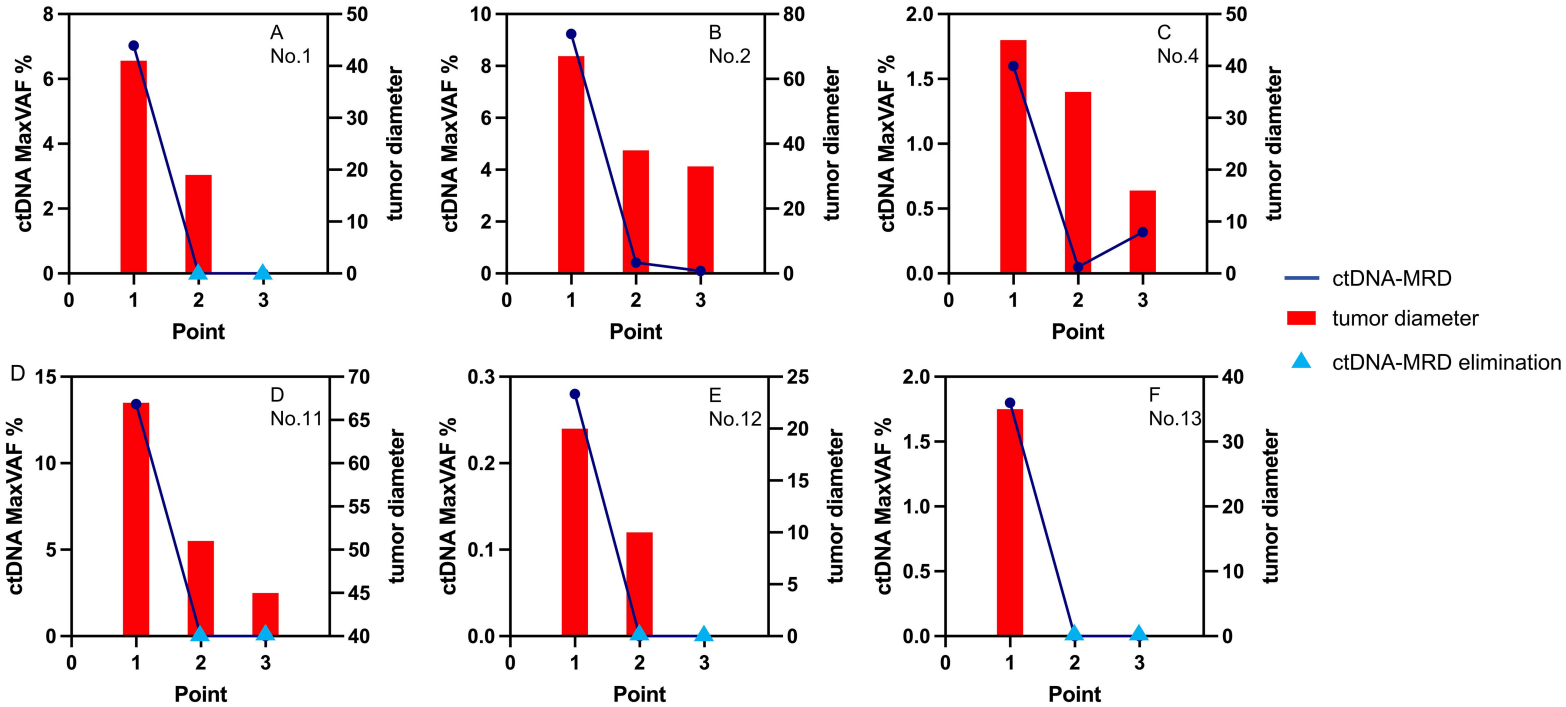
The changes of ctDNA and tumor diameters in the 4-cycle group. ctDNA, circulating tumor DNA; MaxVAF, maximal tumor somatic variant allelic frequency; tumor diameter, measured by millimeter.

As shown in **Table 2**; **Figure 5**, in the four-cycle NCI group, all cases had tumor diameter decreasing, and all cases except Case No.4 had ctDNA-MRD decreasing. The accordance rate of the trends was recorded as 83.3% (5/6). As to Case No.4, although the tumor kept shrinking (clinically PR), ctDNA-MRD had a small jump in the process. It might indicate the poor pathological outcomes (ypT1cN1, not-MPR).

## Discussion

In this prospective study, 13 patients with 28 peripheral blood samples were included. To the best of our knowledge (9), it was the first one that explored the association between ctDNA-MRD and pCR and neoadjuvant cycles. It showed some other clinical and pathological outcomes that also deserved further discussion.

The observation in the two-cycle group suggested that two-cycle NCI alone could bring about pCR, this was consistent with the findings from other clinical studies (9, 14). But this pCR rate was obviously lower than that in the counterpart (14.2% versus 50.0%). Given the vital role of pCR, additional NCI cycles seemed to be necessary. On this basis, we further explored whether ctDNA-MRD could work as an indicator of the potential pathological benefit of additional NCI cycles.

We found a particular association between ctDNA-MRD and pCR: ctDNA-MRD elimination was not always followed by subsequent pCR, while pCR only occurred among those with ctDNA-MRD elimination. In other words, this seemed to imply that ctDNA-MRD elimination was a necessary but not sufficient condition for pCR. It was an interesting and potentially valuable deduction. As a small-sample study, it could not be drawn out as a definitive conclusion. However, it was theoretically consistent with tumor biology. To a certain degree, even if this deduction was not true, pCR with ctDNA-elimination could be a subgroup different from pCR without ctDNA-elimination. A large-sample study would be helpful to offer reveal insights.

The observation in the four-cycle group indicated that if ctDNA-MRD elimination was not achieved after the initial two-cycle NCI, it would not be achieved after the additional two-cycle NCI. When this was taken into consideration with the above presumption, it could be further summarized that if ctDNA-MRD elimination was not achieved after the initial two-cycle NCI, ctDNA-MRD elimination, together with pCR, would not be achieved after the additional two-cycle NCI. This sounded appealing. But similarly, more data was needed to validate it. We reasoned that the two-cycle NCI was enough to test tumor sensitivity (even bring about pCR), and the additional enhanced regimen could not overcome tumor resistance. AEGEAN study had suggested that patients who achieve ctDNA clearance early during neoadjuvant therapy were more likely to attain higher pCR and MPR rates, indicating that ctDNA clearance might serve as a potential early biomarker of treatment response (23). From this perspective, if ctDNA-MRD elimination was not achieved after the initial two-cycle NCI, would a changed regimen work better?

Of all the patients, although the blood samples were collected before every cycle and before the surgery, the ctDNA-MRD was detected at two-cycle intervals because of the cost. We believed that a more frequent detection would offer more information, especially about the third and fourth cycles. Although an additional two-cycle NCI made a higher pCR rate, it might also cause more adverse events. In this study, treatment toxicities were not shown and discussed. A biomarker effectively predicted pCR would be valuable as it maximized benefits and minimized harms. We expected ctDNA-MRD could be a candidate.

This study has some limitations. First, although this was a prospective randomized controlled clinical study, the sample size was too small to draw definitive conclusions, let alone guided clinical practice. It merely gave some insights into an important but seldom explored issue. Second, due to economic reasons, the detection was not conducted for every cycle and this could not reveal a real ctDNA-MRD trend. Third, this study explored the association with pathological outcomes rather than survival outcomes. Although pCR was sometimes accepted as a surrogate endpoint, this still limited the clinical value.

## Conclusions

According to the study, patients with locally advanced NSCLC who achieved ctDNA-MRD elimination after two-cycle NCI were more likely to benefit from additional two-cycle NCI, manifesting higher pCR rates. ctDNA-MRD could be a promising tool to determine the optimal cycle of NCI.

## Data Availability

The original contributions presented in the study are included in the article/**Supplementary Material**. further inquiries can be directed to the corresponding authors.
